# Are complex traits underpinned by polygenic molecular traits? A reflection on the complexity of gene expression

**DOI:** 10.1093/pcp/pcae140

**Published:** 2024-11-29

**Authors:** Mohsen Hajheidari, Shamil Sunyaev, Juliette de Meaux

**Affiliations:** Institute for Plant Sciences, Cluster of Excellence on Plant Sciences (CEPLAS), University of Cologne, Cologne 50674, Germany; Department of Biomedical Informatics, Harvard Medical School, Boston, MA 02115, USA; Institute for Plant Sciences, Cluster of Excellence on Plant Sciences (CEPLAS), University of Cologne, Cologne 50674, Germany

**Keywords:** Complex traits, Polygenic, Gene expression, Transcription factors, Regulatory elements, The Mediator complex, Chromatin modifiers, Transcription factor-DNA binding

## Abstract

Variation in complex traits is controlled by multiple genes. The prevailing assumption is that such polygenic complex traits are underpinned by variation in elementary molecular traits, such as gene expression, which themselves have a simple genetic basis. Here, we review recent advances that reveal the captivating complexity of gene regulation: the cell type, time point, and magnitude of gene expression are not merely dependent on a couple of regulators; rather, they result from a probabilistic process shaped by *cis*- and *trans*-regulatory elements collaboratively integrating internal and external cues with the tightly regulated dynamics of DNA. In addition, the finding that genetic variants linked to complex diseases in humans often do not co-localize with quantitative trait loci modulating gene expression, along with the role of nonfunctional transcription factor (TF) binding sites, suggests that some of the genetic effects influencing gene expression variation may be indirect. If the number of genomic positions responsible for TF binding, TF binding site search time, DNA conformation and accessibility as well as regulation of all *trans*-acting factors is indeed vast, is it plausible that the complexity of elementary molecular traits approaches the complexity of higher-level organismal traits? Although it is hard to know the answer to this question, we motivate it by reviewing the complexity of the molecular machinery further.

## Introduction

Traits showing quantitative variation are often controlled by both environmental factors and multiple variants in the genome. Such complex traits are central in evolutionary biology and breeding programs. The genetic variation they display in natural populations depends on the opportunities along the genome for sequence changes that have an effect on the trait. Large effect alterations in single genes can be instrumental for modifying complex traits in systems as diverse as, e.g. the crop *Zea mays*, the model plant *Arabidopsis thaliana*, the natural plant species *Boechera stricta*, or the long-lived balsam poplar *Populus balsamifera* (Studer et al. 2011, Keller et al. 2012, Prasad et al. 2012, Tergemina et al. 2022). Yet, the dissection of the molecular pathways underpinning these traits indicates that they rely on the action of multiple genes and pathways, expressed in different organs or environmental conditions. The number of positions in the genome that, when changed, can modify a given trait is called the mutational target size, and it is a key parameter that defines the amount of genetic variation the trait is expected to display (Hollinger et al. 2023).

All cells of multicellular organisms share the same DNA, yet the transcriptional activity of genes in distinct cell lineages allows a diversity of tissues and organs to form. Since the groundbreaking work of Jacob and Monod in 1961, which unveiled the existence of regulatory DNA sequences, gene regulation and its variations have been assumed to be relatively straightforward (Nora et al. 2023). This idea, centered on the paradigm of a transcription factor (TF) interacting with cognate TF binding sites (TFBSs), has long dominated our understanding of regulatory variation: an oligogenic trait regulated by a small number of *cis*- and *trans*- variants (Wray et al. 2007, Wittkopp 2023). It is therefore usually assumed that complex traits are underpinned by simpler traits, such as gene expression levels (Cookson et al. 2009). However, as we will detail in this review, many proteins and protein complexes are involved in the expression of any single gene in multicellular eukaryotes, including in plants. These include sequence-specific TFs, basal/general TFs, chromatin modifiers, the Mediator complex, and RNA polymerase II (RNAPII) (Haberle and Stark 2018, Richter et al. 2022, Bieluszewski et al. 2023). Since each of these proteins and protein complexes can vary, the genetic basis of variation in gene expression can be much more complex than initially thought. In light of this complexity, it appears that many variants in the genome can alter gene expression. In such context, it becomes harder to view gene expression as the simple oligogenic variance that underpins complex polygenic traits. We argue that future studies will have to consider that complex traits may well be controlled to be a suite of underpinning traits of comparable complexity.

## Regulatory Regions of the Genome

**Figure 1.**
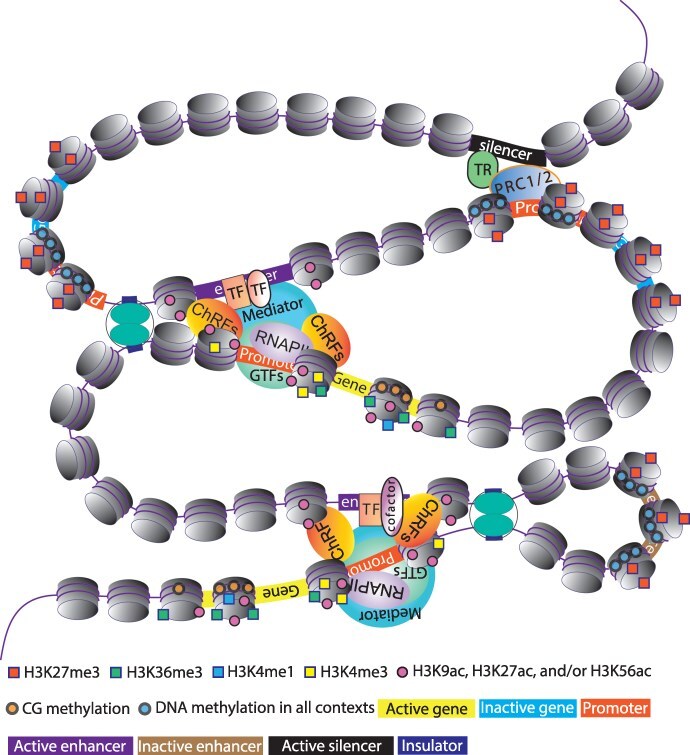
A model of transcriptional regulation in plants. TFs specifically bind to their corresponding binding sites within *cis*-regulatory elements, including enhancers and silencers, which are located in accessible chromatin regions. The binding of an activating TF initiates a cascade of events, culminating in the recruitment of chromatin-remodeling factors (ChRFs), the Mediator complex, general/basal TFs (GTFs), and RNAPII at the target loci. Transcriptional repressor (TR) represents a transcriptional factor that binds to the silencer and inhibits transcription of the target gene by recruiting the PcG complexes (PRC1/PRC2). Epigenetic patterns are represented according to the activity/inactivity of genes and enhancers. DNA methylation in all contexts depicts DNA methylation in three sequence contexts (CG, CHG, and CHH, with H representing any nucleotide except G). CG methylation depicts DNA methylation in CG context. H3K9ac, histone H3 acetylation at lysine 9; H3K27ac, histone H3 acetylation at lysine 27; H3K56ac, histone H3 acetylation at lysine 56; H3K36me3, histone H3 trimethylation at lysine 36; H3K4me1, histone H3 monomethylation at lysine 4; H3K4me3, histone H3 trimethylation at lysine 4; H3K27me3, histone H3 trimethylation at lysine 27. Histone methylation and acetylation marks are represented by squares and circles, respectively.

The molecular mechanisms that govern development, homeostasis, and responses to environmental stimuli rely on the intricacies of transcriptional regulation. At the core of transcriptional regulation are *cis*-regulatory elements (CREs), which include promoters, enhancers, silencers, and insulators (Fig. 1). Their functionality is intertwined with the TFs they interact with and the regulators of the genomic context in which they operate. Understanding CREs is crucial because as evolutionary distance increases, so does the divergence of CREs driving the genetic alterations that underpin the evolution of complex traits (Metzger et al. 2017, Osada et al. 2017).

### Core promoters integrate basal TFs, the Mediator complex, and gene-specific regulatory elements

Promoters, due to their predictable positions near gene transcription start sites (TSSs), constitute the most extensively studied category of the CREs. To unravel how they enable cell- and environment-specific gene expression, we must examine the intricate interplay between signals orchestrating the recruitment of the transcription machinery and the promoter-specific elements.

The chromatin around promoters remains accessible, thanks to the presence of the unstable histone variant H2A.Z. These accessible core promoters act as initiation sites for transcription, facilitate the assembly of the transcriptional machinery, and enable basal transcription (Haberle and Stark 2018). Core promoters can be classified as narrow or broad (Yamamoto et al. 2011). Narrow promoters initiate transcription from a single TSS or a distinct cluster of TSSs over a few nucleotides. In contrast, broad promoters contain several start sites, up to 50 to several 100 nucleotides. Narrow promoters are often associated with developmental and tissue-specific genes, while broad promoters are often associated with constitutively expressed genes characterized by ubiquitous, low-level expression (Lenhard et al. 2012, Mejía-Guerra et al. 2015). In eukaryotes, the core promoter may contain a TATA box, an initiator (Inr) element, and a downstream promoter element (DPE) (Ngoc et al. 2017). The Inr elements surround the TSS, whereas the DPEs are located downstream of the TSS (Ngoc et al. 2017). Specific core promoter elements may be unique to certain eukaryotic groups, such as the plant-specific Y-patch element or the CpG islands found in many human core promoters (Yamamoto et al. 2011). The motif ten element, found in metazoans, is often situated ∼18–30 base pairs downstream of the TSS (Theisen et al. 2010). The core promoter elements position RNAPII at the precise TSS, ensuring the initiation of accurate transcription. Basal TFs, RNAPII, and other regulatory proteins must recognize these elements for the preinitiation complex (PIC) to be formed; recognition marks the onset of transcriptional activity for a given gene (Patel et al. 2018, Richter et al. 2022). In addition, chromatin epigenetic modifications at the +1 nucleosome in the promoter region, such as the acetylation of histone H2A.Z and trimethylation of histone 3 lysine 4 (H3K4me3), common in actively transcribing genes, can enhance transcription (Gomez-Zambrano et al. 2019). Conversely, the DNA methylation of promoter regions represses transcription (Le et al. 2020). The combinations and arrangements of core promoter elements vary among genes and contribute to the mechanisms that regulate gene expression in eukaryotic organisms in the context of different tissues and environmental conditions. The variable structure of light-regulated promoters illustrates this phenomenon in plants (Srivastava et al. 2014).

To initiate transcription, the promoter recruits basal TFs as well as a protein complex, called the Mediator complex, thereby completing the PIC. The Mediator complex links basal transcription mechanisms to gene-specific activating factors. The TF II D (TFIID) complex assembles the PIC on the core promoter, with the help of the Mediator complex, through a series of intricate interactions; these interactions have been well studied in yeast and metazoans (Chen et al. 2022, Richter et al. 2022). TFIID, comprising the TATA-binding protein (TBP) and TBP-associated factors (TAFs), is the first factor to bind to the core promoter. With multiple DNA-binding domains, TFIID recognizes various core elements in different promoters, facilitating TBP loading onto diverse core promoters, such as TATA-containing and TATA-less promoters (Patel et al. 2018). The Mediator complex and TFIID collaborate to position the polymerase RNAPII within the PIC (Richter et al. 2022). TFIIA and TFIIB are additional complexes subsequently recruited to opposite sides of the TFIID subunit TBP. TFIIA plays a stabilizing role in the binding of TFIID to the promoter, whereas TFIIB identifies the TFIIB recognition element in promoters and directly interacts with RNAPII (Kostrewa et al. 2009). Following the binding of TFIIB, TFIIF is recruited, forming a stable complex at the promoter (Thomas and Chiang 2006). This event drives the association of TFIIE and the subsequent entry of TFIIH that is positioned within the PIC by the Mediator complex (Compe et al. 2019). Like TFIIB, TFIIE and TFIIF bind directly to the RNAPII (Richter et al. 2022). The Mediator complex integrates upstream activators with the PIC. It stimulates the RNAPII C-terminal domain kinase activity of cyclin H-dependent kinases. As a result, the Mediator complex is released from RNAPII, coinciding with the enrichment of the phosphorylated Ser5 (Ser5P) C-terminal domain mark. This phosphorylation event facilitates promoter clearance and marks the transition from transcription initiation to transcription elongation (Hajheidari et al. 2013). The multimolecular process necessary to initiate transcription demonstrates how the basal activation of gene expression is controlled by a polygenic molecular basis.

Beyond the mechanism of basal gene expression activation, the magnitude of gene expression must also be regulated, in a process that reflects the specificity of each gene and each cellular context. To this end, the activation of the core promoter must be connected with the regulatory signals provided by CREs. The Mediator complex fulfills this role. It is a large assembly, comprising three core modules—the head, middle, and tail—and a separable regulatory cyclin-dependent kinase 8 module. Notably, the tail module exhibits significant structural flexibility, facilitating interactions with sequence-specific TFs and co-factors. It is such interactions that form a bridge between *cis*-regulatory elements and the basal transcription machinery. Interestingly, Mediator subunits in plants have expanded and diversified: individual knockouts of Mediator subunits in *A. thaliana* are not lethal; they alter growth and development or impair responses to both biotic and abiotic cues (Chen et al. 2022). As a result, the composition of the Mediator complex can be variable in plants, and this variation, coupled with the flexibility it offers for interactions with TFs and co-factors, contributes to gene-specific transcriptional regulation (Chen et al. 2022, Freytes et al. 2024).

### Enhancers shape gene expression

Enhancers play a critical role in cell type– and tissue-specific gene expression and contribute to gene regulation in response to environmental cues (Jores et al. 2024). Unlike promoters, enhancers lack the capability to independently initiate transcription. To streamline transcription, they must be brought close to promoters by DNA loops formed, thanks to the three-dimensional structure of chromatin (Haberle and Stark 2018). The discovery of the first enhancer was reported by Banerji et al. in 1981. In their study, remarkably, a 72-base-pair (bp) repeat from simian virus 40 (SV40) genome was able to enhance the expression of the human β-globin gene when positioned at various locations relative to the gene promoter. Enhancers can be found upstream, downstream, or even within introns of their target genes, spanning distances from a few kilobases in plants and *Drosophila* to nearly a megabase in mammals (Vlad et al. 2014, Vuolo et al. 2016, Field and Adelman 2020). Distal enhancers are found in multicellular organisms with large genomes more frequently than in multicellular organisms with small genomes, such as the model plant *A. thaliana* (Maher et al. 2018, Lee and Seo 2023). Enhancers can operate irrespective of their orientation (Jores et al. 2024). These short DNA sequences, typically 50–1500 bp, contain clusters of binding sites for sequence-specific TFs. When a group of typical enhancers is located close together in the genomic sequence, they form superenhancers; these are likely instrumental in generating various cell types by driving expression during tissue formation. Notably, superenhancers can extend to 100 kb. In *A. thaliana*, a set of 729 putative superenhancers has been identified (Zhao et al. 2022). The architecture of *cis*-regulatory elements within enhancers is complex, encompassing various factors such as the type of TF binding motifs; the number and affinity of binding sites; DNA shape features; and the spacing, order, and orientation of binding motifs (Junion et al. 2012, Hajheidari and Huang 2022). This intricate arrangement ultimately determines the functional output of enhancers.

Enhancers exhibit a wide spectrum of architectures, yet three commonly employed models simplify their characterization. In instances where a strict and organized arrangement of TFBSs within the enhancer is crucial for the proper recruitment of transacting factors—e.g. a fixed combination, order, spacing, and direction of binding sites—the enhancer is classified under the “enhanceosome” model. On the other hand, when there are limited organizational constraints on TFBSs, and not all binding sites are active simultaneously, the enhancer aligns with the “billboard” model. This model allows for a degree of tolerance toward decreased or altered TFBS affinities. Such tolerance, coupled with positional flexibility, contributes to a higher rate of turnover in binding sites or slight conformational alterations in TFs during evolutionary processes across species. When a specific combination of TFs is necessary for enhancer activation—not all TFs must bind directly to the DNA and instead are engaged via protein–protein interactions—the enhancer aligns with the “TF collective” model (Junion et al. 2012).The three enhancer models differ in how strict the relationship is between binding sites and the TFs that are involved in initiating transcription. Such three models highlight the large range of effects that nucleotide substitution in enhancer regions can have on the expression of target genes.

### Silencers

The specific gene expression profile associated with a particular cell lineage is the result of the complex interplay between transcriptional activation and repression (Ngan et al. 2020). Silencers play pivotal roles in the development of organisms, akin to enhancers. These regulatory DNA elements contain TFBSs that actively diminish the expression of target genes by recruiting transcriptional repressors and repressive factors in a tissue- or cell type–dependent manner (Pang et al. 2023). Like enhancers, silencers exhibit modularity. If some regulatory elements are removed, their target genes are activated or ectopically expressed (Hajheidari et al. 2019b, Pang et al. 2023). Like enhancers, silencers operate independent of position and orientation. Recent reports in metazoans systematically surveyed silencers across genomes and revealed that many regions identified as silencers are bifunctional; in other words, they can function as enhancers depending on the cellular context (Ngan et al. 2020, Pang et al. 2023). The results of these studies suggest that silencers consist of multiple subclusters, and thus there are likely no common epigenetic mark/marks or chromatin properties that can be used to universally define silencers (Pang et al. 2023). Although no comprehensive genome-wide report on the identification of silencers in plants currently exists, it is likely that variation in silencers also contributes to the diversification of expression levels. In the rice genome, high-resolution 3D genome maps demonstrated that variation in silencer-like elements enriched with H3K27me3 methylation modulates the downregulation of distal genes (Ouyang et al. 2023).

### Insulators

Gene expression, like many other processes such as DNA replication, recombination, cell division, or stress reactions, depends on the chromatin structure (Golicz et al. 2020, Boltsis et al. 2021). In tomato, the dynamic formation and/or stability of promoter–enhancer interactions during heat stress depends on HSFA1a, a TF essential for heat stress tolerance (Huang et al. 2023). In general, the interplay between chromatin dynamics and environmental cues exemplifies how ecological conditions intersect with genomic processes (regulation of gene expression) to shape a plant’s development or survival strategy.

The influence of genomic architecture operates within a hierarchical framework encompassing chromosome territories (CTs, the spatial arrangement of chromosomes within the nucleus in interphase), chromosome compartments (chromosomes consist of two compartments, one with the active genes in euchromatic regions and the other with inactive genes in heterochromatic regions, also referred to as Compartments A and B, respectively), topologically associating domains (TADs, three-dimensional genomic regions partitioning the genome into distinct regulatory territories), and chromatin loops (Kumar et al. 2021a, Lee et al. 2022). Insulators demarcate TADs, the foundational units of chromatin organization.

The CCCTC-binding factor (CTCF) plays a key role in structuring chromatin in metazoans. CTCF interacts with the cohesin complex and binds to insulator elements, facilitating the formation and maintenance of chromatin loops and ultimately shaping TADs. TADs, in turn, interact to construct chromatin compartments, which merge to constitute CTs (Kurbidaeva et al. 2021, Lee et al. 2022). It is noteworthy that plants, despite lacking an equivalent to the CTCF protein, exhibit analogous three-dimensional chromatin structures, suggesting evolutionary convergence in chromatin organization (Wang et al. 2015, Peng et al. 2019). The significance of these insulating domains will depend on genome size. In plants with compact genomes, such as *A. thaliana*, TADs are not a prominent feature. TAD-like structures have primarily been identified in plants with large genome size: e.g. rice, maize, and tomato. In the rice genome, TADs cover ∼69.7% of the genome, and the median size of a TAD in rice is 35 kb (Golicz et al. 2020). In plants, the presence of plant *cis*-regulatory elements with authentic insulator activity remains uncertain (Wang et al. 2015, Liu et al. 2017). However, research in the plant kingdom has identified sequence motifs enriched at the TAD boundaries for a few TFs, particularly those from the TCP family, but understanding the mechanistic factors orchestrating chromatin organization at the domain level remains a focus of investigation (Liu et al. 2017, Kurbidaeva et al. 2021).

By enabling the formation of chromatin loops, insulators contribute to the regulation of gene expression. In *A. thaliana*, despite the absence of clearly characterized insulators, chromatin around the FLOWERING LOCUS C (*FLC*) gene forms a loop that is thought to facilitate the transcriptional activation of this floral repressor. On the other hand, chromatin architectural factors serve to inhibit looping, and it is a balance between these two processes that ensures correct flowering time (Zhao et al. 2021).

## DNA De-chromatinization Is Required for Both Gene Regulation and Gene Expression

Nucleosomes, the fundamental chromatin units, consist of DNA wrapped around eight histone proteins comprising two copies of histone H3, H4, H2A, and H2B. The tails of these histones can be post-translationally modified by histone modifiers to influence DNA-based processes. Nucleosomes are separated by linker DNA, which interact with so-called linker histones H1. Distinct H1 histone variants bind to the entry and exit sites of DNA on the nucleosome core particles, stabilizing the chromatin structure and contributing to the regulation of transcription (Malik and Henikoff 2003, Sheikh et al. 2023).

The dense compaction of chromatinized DNA tends to hinder the free binding of TFs to specific sites within chromatinized enhancers. Indeed, the binding of TFs to enhancers is generally accompanied by a local depletion of nucleosomes. Approaches such as DNase-seq, FAIRE-seq, ATAC-seq, or MNase-seq couple next-generation sequencing with various marks of active chromatin to predict active enhancers (Hajheidari and Huang 2022). These approaches, however, often overestimate the number of enhancers (Field and Adelman 2020).

Several studies indicate that a subset of TFs, known as pioneer TFs, can bind directly to the nucleosomes *in vitro* (Mayran et al. 2018, Zaret 2018). They can either independently open the closed chromatin or recruit chromatin remodeling factors (Zaret 2018). By doing so, they facilitate the binding of other sequence-specific TFs, initiating cell-specific transcription programs. However, conclusive *in vivo* evidence for active chromatin remodeling by pioneer factors is lacking. This knowledge gap exists primarily because the complex composed of DNA, a TF, and core histone proteins has not yet been observed in living cells (Klemm et al. 2019). Furthermore, the mechanism of action of pioneer TFs remains poorly understood and a subject of debate. The prevailing concept suggests that pioneer TFs bind to transiently accessible DNA, displacing histones passively, rather than actively removing core histone particles from truly histone-bound DNA (Klemm et al. 2019). Additionally, a recent study has indicated that like pioneer TFs, nonpioneer TFs can open inaccessible regions and activate target genes (Hansen and Cohen 2022). These new results challenge the binary categorization of TFs as pioneer and nonpioneer, suggesting instead that “pioneer” is a quantitative trait influenced by the genomic environment, TF concentration, and binding affinity (Hansen and Cohen 2022).

## Chromatin-modifying Factors Modulate the Chromatin Landscape that Conditions the Expression of Each Gene

Chromatin modifiers, remodelers, and histone variants jointly regulate gene expression by altering chromatin density and controlling access to DNA (Pajoro et al. 2014, Samo et al. 2021). The major chromatin-modifying factors include DNA methyltransferases (DNMTs), histone deacetylases (HDACs), histone acetyltransferases (HATs), histone methyltransferases (HMTs), histone demethylases (HDMs), chromatin remodelers, and histone variants. As we detail later, the transcription process is multilayered and integrates signals from DNA methylation, histone modifications, and histone replacements. The dynamics of these elements is controlled by many genes with effects that can alter gene expression both globally and specifically.

### DNA methylation modulates the accessibility of chromatin to the transcription machinery

DNA methylases mainly add methylation residues to cytosines. They have diversified in eukaryotes through independent duplications, gene losses, and sequence divergence (Ponger and Li 2005, Jurkowski et al. 2011). In plants, cytosine DNA methylation is commonly classified according to categories based on the sequence context of the methylated cytosine: CG, CHG, and CHH, with H representing any nucleotide except G (Law and Jacobsen 2010). DNA methylation in repetitive DNA sequences found in both euchromatic and heterochromatic regions is enhanced via a process called RNA-directed DNA methylation (RdDM). RdDM involves two key enzymes, DOMAINS REARRANGED METHYLTRANSFERASE 1 (DRM1) and DOMAINS REARRANGED METHYLTRANSFERAS 2 (DRM2) (Matzke and Mosher 2014, Cuerda-Gil and Slotkin 2016). In addition, plants contain methylases such as METHYLTRANSFERASE 1 (MET1), which is responsible for maintaining CG methylation, and plant-specific CHROMOMETHYLASE 3 (CMT3) and CMT2, which are responsible for maintaining non-CG methylation (Law and Jacobsen 2010, Matzke and Mosher 2014). Recent studies indicate that factors involved in the canonical RdDM pathway predominantly participate in the DNA methylation of already methylated regions, such as heterochromatic regions. However, noncanonical RdDM pathways also initiate DNA methylation in previously unmethylated target loci, while simultaneously contributing to the maintenance of existing methylation. Additionally, it is important to note that RdDM is the only mechanism capable of methylating cytosines, regardless of their sequence context (Cuerda-Gil and Slotkin 2016, Erdmann and Picard 2020).

The methylation of DNA, in turn, shapes various genomic processes including gene regulation, imprinting, and the suppression of transposable elements (TEs); such methylation ultimately affects variation in complex traits such as pathogen defense, stress tolerance, and aspects of plant development (Law and Jacobsen 2010, He et al. 2022, Mattei et al. 2022). For instance, increased DNA methylation is often associated with chromatin compaction and the reduced accessibility of chromatin for TFs and their associated chromatin-remodeling complexes (CRCs) (Kribelbauer et al. 2020). Conversely, hypomethylation of DNA tends to enhance chromatin accessibility. A recent study in *A. thaliana* suggests that local deficiency in DNA methylation generally results in increased chromatin accessibility and enhanced long-range chromatin interactions, only if methylation is absent in all three sequence contexts (Zhong et al. 2021).

Yet, increased chromatin accessibility is not always accompanied by increased transcription. Changes in DNA methylation mediate important ecological responses. Such changes can modulate the expression levels of stress-responsive genes, thereby enhancing plants’ ability to adapt to stresses and possibly contributing to establish a memory of past stress events (Mayer and Charron 2021, Wilkinson et al. 2023). For example, Xie et al. (2019) demonstrated that a reduction in DNA methylation levels within the promoter and coding sequence of *A. thaliana inducer of CBF expression 1* (*AtICE1*), a transcriptional regulator of *C-repeat (CRT)-binding factor* (*CBF*) genes, is associated with increased cold tolerance. This phenomenon is attributed to the enhanced expression of genes within the CBF pathway and enabled by the decreased DNA methylation in *AtICE1* (Xie et al. 2019). DNA methylation can also regulate interactions between alleles. For example, differences in S-allele-specific (self-incompatibility allele-specific) transcript levels in developing flower buds are associated with interallelic small RNA targeting (Burghgraeve et al. 2020) and possibly with DNA methylation (Shiba et al. 2006). Additionally, a truncated duplicate gene that has been translocated to a different chromosome can silence its distant paralog by RdDM, causing incompatibilities (Durand et al. 2012).

### Histone modifiers enable chromatin dynamics

Post-translational modifications of histones are integral to the dynamic nature of chromatin. Numerous studies of plants’ reactions to adverse conditions underscore the significance of reversible and rapid changes in histone methylation and acetylation (Kumar et al. 2021b, Hu and Du 2022, Cui et al. 2023). Active chromatin regions are marked not only by their low level of DNA methylation but also by histone H3 trimethylation at lysine 4 (H3K4me3), H3K36me2/3, and/or high levels of acetylation on histones H3 and H4. Conversely, silent chromatin regions often exhibit histone H3 methylation at lysine 9 and/or 27 (H3K9me and/or H3K27me) (Liu et al. 2010).

As for DNA methylation, histone methylation mobilizes a complex set of proteins. It is catalyzed by three distinct protein families: DOT1/DOT1L, PRMTs, and SET (suppressor of variegation, enhancer of zeste, and trithorax) domain–containing proteins (Hajheidari et al. 2019a). The first, disruptor of telomeric silencing 1–like (DOT1L), is present in mammals and yeast, but absent in plants (Liu et al. 2010). As a consequence, plants lack methylation histone three lysine 79 (H3K79). The second, arginine methyltransferases (PRMTs), catalyzes the addition of one or two methyl groups to the guanidino nitrogen atoms of arginine residues on target proteins; this family plays a crucial role in plant development (Liu et al. 2010). The third group of proteins, the SET domain protein superfamily, comprises all known lysine-specific HMTs and is categorized into seven distinct families (Ng et al. 2007, Fang et al. 2021). In *A. thaliana*, Class I includes enhancer of zeste [E(Z)] homologs responsible for depositing H3K27me2/3. Class II, known as the absent, small, or homeotic disc 1 (ASH1) group, encompasses ASH1 homologs (ASHH) and ASH1-related proteins (ASHR), which catalyze H3K36 methylation. Class III, the trithorax groups (TrxG), including TRX homologs and TRX-related proteins, is involved in catalyzing H3K4me2/3. Class IV, consisting of *A. thaliana* trithorax-related 5 (ATXR5) and ATXR6, exclusive to yeast and plants, is responsible for depositing H3K27me1. Class V, the SU(VAR)3–9 subgroups, includes SU(VAR)3–9 homologs (SUVHs) and SU(VAR)3–9-related proteins (SUVR), catalyzing H3K9 methylation. Classes VI (SMYD) and VII (SETD), comprising proteins with split SET domains, are responsible for the methylation of both histone and nonhistone proteins. Histone modifiers are thus encoded by a large group of genes, whose protein products interact to modulate histone methylation and chromatin compaction.

### Histone methylases and the regulation of transcription

SET domain–containing methyltransferases participate in diverse protein complexes that help integrate environmental cues, such as day length, temperature, and soil moisture; these cues in turn regulate the expression of specific genes during development. A prime example is the polycomb group (PcG) complexes that control developmental transitions in plants. These complexes mediate transcriptional repression through the incorporation of H2A121ub and H3K27me3 by polycomb repressive complexes 1 (PRC1) and 2 (PRC2), respectively (Hinsch et al. 2021). In *A. thaliana*, PRC1 and PRC2 can form multiple subcomplexes with different core and accessory subunit compositions. For instance, an *A. thaliana* PRC2 complex includes the HMTs CURLY LEAF (CLF), SWINGER (SWN), or MEDEA (MEA). These enzymes, which serve as the catalytic subunit of PRC2, are responsible for the trimethylation of histone H3 at lysine 27 (H3K27me3) (Hajheidari et al. 2019a). A recent study by Yin et al. (2021) analyzed chromatin accessibility, histone marks, and gene expression in various *A. thaliana* PcG mutants; chromatin regions marked by H2AK121ub, located at regulatory hotspots, were found to be less accessible than other regions, but they still permitted activator binding, suggesting their importance in transcriptional responses (Yin et al. 2021). In contrast to PcG, TrxG proteins mediate transcriptional activation by catalyzing the methylation of H3K4. For example, ULTRAPETALA1 (ULT1), a trxG factor, physically associates with *A. thaliana* homologs of TRITHORAX1 (ATX1) and promotes the expression of AGAMOUS (AG) and APETALA3 (AP3) genes by both enhancing H3K4me3 deposition and limiting CLF-dependent H3K27me3 deposition (Tyler et al. 2019).

Proteins of the trxG group are also necessary for the regulation of crucial complex traits, such as flowering time, whose variation in plants is shaped by natural selection (Munguía-Rosas et al. 2011). Much of the variation in *A. thaliana* and other relatives is due to variation in two major regulatory genes: FLC and FRIGIDA (FRI) (Kuittinen et al. 2008, Hepworth et al. 2020). FLC is an MADS-box TF that blocks flowering by inhibiting genes required for the transition from vegetative to floral development (Kerbler and Wigge 2023). FLC expression is influenced by FRI and other pathways (Choi et al. 2011, Kerbler and Wigge 2023). TrxG proteins, such as ATXR7 and ASHH2, incorporate H3K4me3 and H3K36 into the FLC locus, whereas FRI acts as a scaffold protein, interacting with other factors to form a transcription activator complex (Choi et al. 2011). This complex recruits general TFs and chromatin modification factors, positively regulating FLC expression (Choi et al. 2011). However, during vernalization, the sequestration of FRI and the deposition of the histone mark H3K27me3 by the cold-induced PRC2 complex progressively repress *FLC* expression (Zhu et al. 2023). This establishment of long-term epigenetic memory at FLC ensures that under favorable conditions, plants transition to the reproductive stage.

Generally, the epigenetic memory encoded by histone modifications allows plants to adjust their development to their environment. Histone modifications, in particular, appear to be a crucial aspect of how plants respond to stress (Baurle et al. 2020). For instance, upon first exposure to drought, a plant acquires a stress memory manifested in the form of the histone mark H3K4me3 on specific stress-responsive genes. This mark maintains the chromatin in a permissive state; remarkably, this memory can persist through many cell divisions, from several days to weeks. This ability allows the plant to respond most effectively to subsequent stress events (Godwin and Farrona 2020).

### HDMs can repress or activate gene expression

The dynamics of chromatin accessibility depend on the balance between histone methylation and demethylation; for balance to be achieved, HDMs are also required. The extent to which the activity of HDMs affects gene expression depends on the type of methylation removed. HDMs are categorized into two groups: lysine-specific demethylase 1 (LSD1/KDM1) and Jumonji C (JmiC) domain–containing proteins (Tsukada et al. 2006). *Arabidopsis thaliana* contains four LSD1-like (LDL) proteins, LDL1-3, and FLOWERING LOCUS D (FLD), all of which catalyze the demethylation of H3K4m1/2. FLD binds to the FLC chromatin and reduces H3K4 methylation with LDL1 and/or LDL2 and contribute to repression of the FLC expression (Jiang et al. 2007). According to sequence similarity, domain organization, and substrate specificity, 21 JmiC domain–containing proteins identified in *A. thaliana* are classified into five subgroups (Chen et al. 2021): KDM4/JHDM3, KDM5/JARID1, JHDM6/JMJD6, KDM3/JHDM2, and JmjC domain–only group. Plants lack the KDM6/UTX/JMJD3 subgroup that is responsible for H3K27 demethylation; however, RELATIVE OF EARLY FLOWERING 6 (REF6), EARLY FLOWERING 6 (ELF6), and JMJ13, which are members of KDM4/JHDM3, work as H3K27 demethylases (Chen et al. 2021, Hu and Du 2022). Plant HDMs play important roles in a wide range of processes, including plant growth and development, seed dormancy, defense responses, and stress responses (Hu and Du 2022). For example, nuclear factor Y (NF-Y) TFs, which form heteromeric complexes, mediate the association of CONSTANS (CO) or DELLAs with the *SUPPRESSOR OF OVEREXPRESSION OF CONSTANS1* (*SOC1*) promoter through the NF-Y subunits. This association leads to reduced levels of H3K27me3 on the *SOC1* locus partly through the recruitment of an H3K27 demethylase REF6 (Hu and Du 2022). In addition, REF6/JMJ12 contributes to thermomorphogenesis by demethylating H3K27 and thereby activating thermoresponsive genes at warm ambient temperatures (He et al. 2022). Histone H3K9me2, linked to gene silencing in plants, is reduced during gene activation induced by drought stress. For instance, the H3K9me1/2 demethylase JMJ27 positively regulates drought responses by influencing genes such as GOLS2 and RD20 (Wang et al. 2021). Under conditions of drought stress, JMJ27 levels increase, directly associating with the chromatin of GOLS2 and RD20. This interaction lowers H3K9me2 levels, inducing gene expression. In fact, alterations in JMJ27 activity affect many complex phenotypes. For example, JMJ27 protects against harmful invaders since it is activated in response to virulent *Pseudomonas syringae* pathogens, and it mediates the cold response by modulating histone methylation in the FLC gene (Hu and Du 2022).

### Histone acetylases place chromatin into a permissive state

Chromatin dynamics and transcriptional regulation are also controlled by HATs. These proteins serve as coactivators that are recruited to the nucleosome by sequence-specific DNA-binding proteins. They transfer an acetyl group from acetyl-CoA to conserved lysine residues of histone proteins to form ε-*N*-acetyllysine (VetteseDadey et al. 1996). Histone tails, which form ∼25% of the mass of isolated histones, contain positively charged residues of lysine and arginine (Wolffe and Hayes 1999). The addition of an acetyl group neutralizes the positive charge of histones, weakening their interaction with DNA. This modification shifts chromatin from a repressive to a permissive state, facilitating access to the DNA for protein factors such as TFs and RNAII (Wolffe and Hayes 1999). HATs exhibit distinct intracellular localizations, which fall into two categories: A-type and B-type. Only the A-type HATs are localized in the nucleus, where they catalyze the acetylation of nucleosome core histones. In *A. thaliana*, A-type HATs have diversified and can be classified into groups based on sequence and structural similarities (Kumar et al. 2021b). These groups include general control nondepressible 5 (GCN5)–related acetyltransferases like HAG1, HAG2, and HAG3; MYST-related HATs represented by HAM1 and HAM2; cAMP-responsive element-binding protein (CREB)–binding proteins (CBPs), including HAC1, HAC2, HAC4, HAC5, HAC12, and TAFs (TAFII250) featuring HAF1 and HAF2. HATs form various complexes, including the SPt-Ada-GCN5-acetyltransferase (SAGA) complex, a sizable multi-subunit assembly conserved from yeast to plants (Wu et al. 2023). The SAGA complex contributes to transcription initiation and elongation by modulating post-translational modifications of nucleosomal histones. In *A. thaliana*, the knockout of the *ADA2b* gene, a core subunit of the SAGA-like complex within the HAT module, leads to diverse developmental defects. These include dwarfing, irregular root development, and reduced petal and stamen length in flowers. Additionally, ADA2b and GCN5 play crucial roles in the plant’s responses to both biotic and abiotic stresses.

Similar to methylation, HAT activity can be reversed to balance chromatin permissivity in different cell types and/or environments. The action of HAT is reversed by HDACs, which allow chromatin to recondense. HDACs are classified into several groups based on sequence and structural similarities. This classification includes reduced potassium dependency 3, which is further divided into three classes: Class I (HDA6, HDA9, and HDA19), Class II (HDA5, HDA8, HDA14, HDA15, and HDA18), and Class III (HDA2). Additionally, it includes histone deacetylase 1 (HDA1); silent information regulator 2, encompassing SRT1 and SRT2; and histone deacetylase 2 (HD2), featuring HD2A, HD2B, HD2C, and HD2D (Shen et al. 2015, Kumar et al. 2021b).

### Chromatin remodelers liberate the energy for the mechanical remodeling of chromatin

While histone modifications can alter the status of chromatin, specific proteins are needed to mechanically regulate its conformation dynamic. The sucrose nonfermenting 2 (Snf2) chromatin-remodeling ATPase family, a member of the helicase superfamily SF2, encompasses several subfamilies (Flaus et al. 2006). Four of these—switch defective/sucrose nonfermentable (SWI/SNF), imitation switch/sucrose nonfermentable 2 like (ISWI/SNF2L), chromodomain helicase DNA-binding (CHD), and inositol requiring 80/SWI2/SNF2-related 1 (INO80/SWR1)—are particularly crucial for transcriptional regulation in accessible euchromatin. These subfamilies have different structures that make them functionally and genetically distinct. CRCs transform the chemical energy derived from the hydrolysis of ATP into mechanical motion. This process includes the sliding of nucleosomes along the DNA helix, the disassembly of the nucleosome, and the exchange of histone variants (Flaus et al. 2006; Hou et al. 2016). Thus, CRCs play a substantial role in regulating DNA accessibility for DNA-binding proteins and co-factors. CRCs play a dual role: they not only regulate stress-responsive genes during stress but also actively suppress them in the absence of stress. This dual role helps optimize resource distribution during plant growth and development (Willige et al. 2021, Bieluszewski et al. 2023). In *A. thaliana*, the SWI/SNF ATPases are represented by BRAHMA (BRM/CHR2), SPLAYED (SYD/CHR3), and two closely related entities, MINUSCULE1 (MINU1/CHR12) and MINUSCULE2 (MINU2/CHR23). The AtSWI/SNF ATPases assemble into large complexes with both similar and different functions (Guo et al. 2022). BRM, a core ATPase subunit of the SWI/SNF chromatin-remodeling complex, contains a helicase/SANT-associated (HAS) domain upstream of the ATPase, which serves as a binding platform for nuclear actin-related proteins (Szerlong et al. 2008). Additionally, BRM has a C-terminal bromodomain that can bind to acetylated lysine residues. The knockout mutation of the SWI/SNF ATPases leads to significant developmental defects, underscoring their global importance. For instance, single mutants (*brm* and *syd*) exhibit a reduction in plant size, slow growth, abnormal development of leaves and flowers, and significantly reduced fertility, and the *brm syd* double mutant displays embryonic lethal phenotypes, with development arrested at the early heart stage (Bezhani et al. 2007). *Arabidopsis thaliana* has two ISWI-type ATPases: CHROMATIN REMODELING11 (CHR11) and CHROMATIN REMODELING17 (CHR17) (Shang and He 2022). AtISWI proteins, which are functionally redundant, form different complexes with the AtDDT (DNA-binding homeobox and different TF)–domain proteins; they also control multiple developmental processes, including vegetative development, flowering time, floral organ identity, and fertility (Li et al. 2014). Proteins belonging to the CHD subfamily exhibit a distinctive structural feature: two closely positioned chromodomains located at the N-terminus. These chromodomains are able to engage with methylated histones and/or DNA (Gentry and Hennig 2014). In *A. thaliana*, four CHD genes have been identified: *PKL, CHR4, CHR5*, and *CHR7* (Gentry and Hennig 2014). CHD remodelers are involved in mRNA processing, as well as transcriptional regulation. *Arabidopsis thaliana* contains INO80 and SWR1/PHOTOPERIOD INDEPENDENT EARLY FLOWERING 1 (PIE1) CRCs. The INO80 and SWR1 complexes, like other chromatin-remodeling assemblies, function as transcriptional regulators. Furthermore, they are involved in the DNA repair system and DNA recombination (Gerhold and Gasser 2014).

In addition to the abovementioned chromatin remodelers, several other chromatin remodelers from Snf2 family also contribute to heterochromatin formation and maintenance via DNA methylation. For example, DECREASED IN DNA METHYLATION 1 (DDM1) and DEFECTIVE IN RNA-DIRECTED DNA METHYLATION 1 (DRD1) maintain DNA methylation, ensure transcriptional silencing, and promote genome stability (Shang and He 2022). Depletion of DDM1 leads to a widespread decrease in DNA methylation and enhances chromatin accessibility.

Chromatin remodelers belong to the global expression machinery, but they are generally recruited by TFs to specific target sites where they locally modulate the accessibility of genomic DNA. For example, PIF3, BZR1, and HY5 TFs channel PKL, which is a chromatin remodeler of the CHD subfamily, toward *cis*-regulatory regions of genes related to cell elongation (Zhang et al. 2014). Under normal conditions, the activation of shade-avoidant or heat-responsive genes is prevented by enrichment in the gene body of H2A.Z. Under conditions of low red/far-red light ratio or elevated temperature, PIFs promote H2A.Z eviction by directly interacting with INO80c, and genes that enhance hypocotyl elongation become active (Willige et al. 2021). The establishment and maintenance of cell identity and organ development via the transcriptional regulation of developmentally important genes also necessitate the coordinated action of TFs, chromatin remodelers, and histone methylases of the PcG complexes. For example, before flower formation, PcG complexes inhibit the expression of the floral homeotic regulators, such as *APETALA3* (*AP3*) and *AGAMOUS* (*AG*) genes, which, crucially, specify the male and female reproductive organs. However, at the onset of flowering, LEAFY (LFY) and SEPATALLA3 (SEP3) TFs counteract the inhibitory function of PcG proteins by targeting the SWI/SNF ATPases SYD and BRM to *cis*-regulatory regions of *AP3* and *AG* (Wu et al. 2012).

### Histone variants replace canonical histones to foster gene expression

Histones are diverse, existing in several nonallelic protein isoforms that can replace canonical histones within nucleosomes. These variants orchestrate histone modifications and chromatin dynamics and affect gene expression in diverse ways (Kumar and Wigge 2010, Dai et al. 2017).

When present on gene bodies, one of these variants, H2A.Z, leads to reduced chromatin accessibility by promoting the deposition of H3K27me3 and the monoubiquitination of H2A.ZK129 through the action of the abovementioned histone methylases PRC2 and PRC1, respectively (Dai et al. 2017, Gomez-Zambrano et al. 2019). Additionally, H2A.Z exerts a negative regulatory effect on enhancer activity by depositing H3K27me3 and removing H3K4me3 histone marks (Dai et al. 2017). Similarly, H2A.Z occupancy at the TSS is typically associated with the absence of gene expression, and its depletion leads to transcription induction. For instance, factors such as an increase in temperature or the inactivation of the REF6 gene (a gene that is crucial for H2A.Z deposition) can result in the depletion of H2A.Z occupancy at the TSS. This depletion, in turn, induces the transcription of certain genes, including temperature-responsive genes and *FLOWERING LOCUS T* (*FT*) gene (Kumar and Wigge 2010, Smith et al. 2010). On the other hand, H2A.Zac has been observed to positively correlate with transcriptional activity (Gomez-Zambrano et al. 2019). Moreover, H2A.Z occupancy may play a crucial role in preventing the silencing of the DNA methylation–mediated gene (Zilberman et al. 2008) and safeguarding against the transition of chromatin to heterochromatin.

Another isoform, the H2A.W histone variant, characterized by an extended C-terminus containing an SPKK motif, is crucial for heterochromatin condensation (Yelagandula et al. 2014). Constitutive heterochromatin marked by H3K9 methylation exhibit enrichment in H2A.W, whereas suppressed genes in facultative heterochromatin primarily bear the H2A.Z mark (Jiang and Berger 2023). In contrast to H2A.Z and H2A.W, the variant H2A.X is predominantly located within the bodies of active genes (Lei and Berger 2020).

The histone variant H3.1 is predominantly enriched in silent regions in *A. thaliana*, such as in repetitive pericentromic heterochromatin, which are preferentially marked by H3K9me2 and H3K27me1. Conversely, H3.3 is mainly found in the promoters and gene bodies of active genes, primarily those marked by H3K36 (Lu et al. 2018). Additional histone variants, including H1.1, H1.2, and H1.3, have been identified among linker histones that bind to DNA-separating nucleosomes. Analysis of mutants for the three canonical variants of *A. thaliana* H1 histones revealed that double *h1.1h1.2* and triple *h1.1h1.2h1.3* mutants exhibited resistance to *Pseudomonas syringae* and *Botrytis cinerea* infections (Sheikh et al. 2023). Changes in H1.3 expression and its deposition in the promoter and/or gene body of responsive genes are believed to contribute to how plants respond to stress (Rutowicz et al. 2015).

## TFs Have to Search for Their Target Sites in the Genome

**Figure 2.**
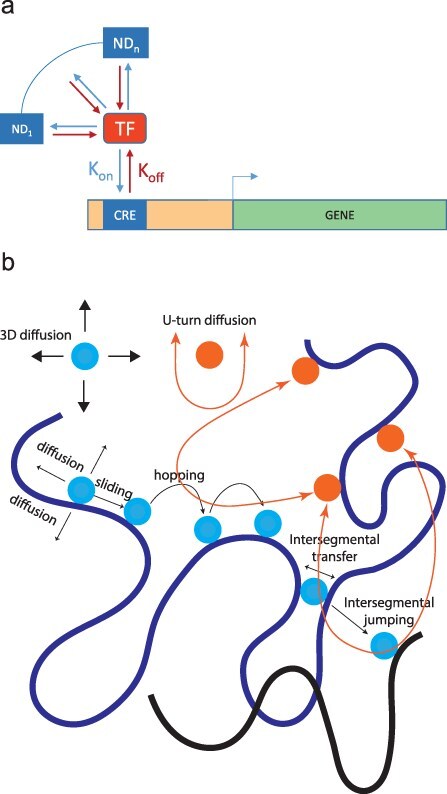
Models for how TFs diffuse, interact with, and navigate their specific binding sites. (a) Dynamic of TF-DNA binding: a TF randomly and transiently associates with its own specific binding site within a *cis*-regulatory element and subsequently dissociates from and likely binds to a nonspecific binding site that works as natural decoy (ND). Afterward, the TF may either bind to another nonspecific binding site or rebind to its original specific binding sites. (b) Based on the facilitated diffusion model, TFs diffuse isotropically, diffusing randomly and freely to all directions, until they collide with a DNA molecule. Upon random collision, TFs establish weak interactions with nonspecific binding sites largely through electrostatic forces. The weak interactions facilitate 1D sliding of TFs along the DNA helix (TFs search their binding sites by combination of 1D sliding with other motions such as hopping, intersegmental transfer, and intersegmental jumping). Although this model has been supported in prokaryotes and *in vitro* eukaryotic data, current technologies lack the capability to provide *in vivo* evidence for local motions such as 1D sliding in eukaryotes. In addition, recent studies demonstrate that some TFs show anisotropy diffusion, making more U-turns than expected by chance. These TFs scan the genome more locally, whereas TFs that show isotropic diffusion search the genome globally.

Advancements in using fluorescence microscopy such as fluorescence recovery after photobleaching, fluorescence correlation spectroscopy, and single-molecule tracking (SMT) provide a mechanistic understanding of how TFs regulate their target genes (Chen et al. 2014). Both weak and strong intermittent interactions between TFs and DNA are needed for TFs to find their target sites. TFs explore the three-dimensional nuclear space and exhibit different movement patterns that determine their search times. Some move isotropically, diffusing freely to all directions, whereas others show anisotropy, making more U-turns than expected by chance (Izeddin et al. 2014). Anisotropic exploration leads TFs to oversample their nuclear neighborhood, whereas isotropic diffusion leads to global exploration and the likelihood that any target can be reached, regardless of distance (Izeddin et al. 2014). If the interaction is nonspecific, TFs typically engage with chromatin for less than a second. Their average residence time, however, increases sharply (from 10 to 100 s) when TFs are bound to specific sites (Mazzocca et al. 2021). According to SMT data, the residence times of a given TF vary across specific binding sites (Mazzocca et al. 2021). Accordingly, active genes do not continuously produce messenger RNAs (mRNAs). Instead, transcription occurs in stochastic bursts that alternate with periods of inactivity (Fig. 2a). Therefore, gene expression levels can be described as probabilistic rather than deterministic (Leyes Porello et al. 2023). The properties of bursting are defined by parameters such as duration, frequency, and size (Dar et al. 2012). Furthermore, because TFs bind to quasi-sequences whose affinities are akin to those of specific binding sites, the distinction between specific and nonspecific binding is complicated (Kemme et al. 2016) (Fig. 2).

TFs need to scan a vast genome before they can locate their target regulatory sequences, which are interspersed within a large amount of nonspecific DNA. Experimental observations have shown that the search time of sequence-specific DNA-binding proteins is significantly faster than estimates based on diffusional collisions between the protein and the DNA molecule (Chen et al. 2014). To address this search problem, the facilitated diffusion model was proposed (Vonhippel and Berg 1989). This model suggests that a combination of at least two dynamic motions can enhance the accuracy and speed of target binding by sequence-specific DNA-binding proteins. In the facilitated diffusion model, 3D diffusion of TFs leads to random collisions with segments of accessible DNA. Upon collision, TFs form weak interactions with nonspecific binding sites, primarily through electrostatic forces. This nonspecific DNA-TF binding facilitates rotation-coupled one-dimensional (1D) sliding along the DNA helix (Vonhippel and Berg 1989, Tafvizi et al. 2011). The reduction from 3D Brownian diffusion to 1D sliding significantly decreases the time required to search for targets. TFs then slide along DNA, in a unidimensional process, until they reach specific target sites and either form stronger interactions, mainly through hydrogen bonds and van der Waals interactions, or dissociate from DNA due to obstacles or their finite dissociation constant. The dissociation of TFs from DNA molecule might be followed by hopping, intersegmental jumping, and/or intersegmental transfer (Tafvizi et al. 2011, Subekti et al. 2020) (Fig. 2b). Although this model has been supported in prokaryotes and *in vitro* eukaryotic data, current technologies lack the capability to provide *in vivo* evidence for local motions such as 1D sliding. Searching for target sites in eukaryotes is likely more complex than for prokaryotes, and various factors contribute to how efficient a search is (Lu and Lionnet 2021). For instance, the majority of all eukaryotic TFs contain intrinsically disordered regions, which mediates TF assembly into clusters (Lu and Lionnet 2021). Clustered binding sites also contribute to the formation of TF clusters, facilitating target site searching through association with specialized compartments (Yan et al. 2013, Lu and Lionnet 2021). In mammals, TF binding primarily occurs in dense clusters around cohesin anchor sites, and thus the majority of TF clusters contain cohesin, which facilitates the maintenance of TF clusters after DNA replication (Yan et al. 2013, Bolanos-Villegas et al. 2017). The genomic landscape of TF-DNA binding is cohesin therefore also influenced by the general processes that affect DNA replication.

## Gene Expression Likely Underpins Complex Traits, But Often in an Indirect Manner

Complex higher-level phenotypes are determined by the joint effect of the suite of molecular, cellular, or developmental phenotypes often called endophenotypes in medicine (Gottesman and Gould 2003). Identifying these endophenotypes and their variation is a necessary step toward understanding the chain of events that shape complex traits. The heritability of complex diseases in humans is enriched in regulatory DNA sequence variants (Maurano et al. 2012, Finucane et al. 2015), suggesting that gene expression might be one of the primary phenotypes (Yao et al. 2020).

Despite the flurry of data and a clear association with variants located in open chromatin, humans have shown few links between emergent variants and gene expression (Maurano et al. 2012, Schaub et al. 2012, Chun et al. 2017; Umans et al. 2021, Yao et al. 2020). For example, genome-wide association studies (GWAS) peaks for 11 common human diseases were shown to be better predicted by the cell type–specific chromatin landscape than by the colocalization with expression quantitative trait loci (eQTLs) obtained from expression analyses in bulked tissues (Gusev 2014). In a quest to understand how trait-associated genetic variants influence gene expression, Connally et al. (2022) pinpointed 220 genes associated with seven common diseases and three quantitative traits. Each of these diseases has a severe form caused by known coding variants. After adjusting for coding variation, the researchers investigated whether these genes are enriched in the vicinity of variants associated with the polygenic form of each trait. They discovered that 147 genes near GWAS signals are likely targets of trait-associated noncoding variants. For most of these genes, they found significant eQTLs. However, their results revealed limited evidence that the baseline expression of these genes explains the associations between complex traits and genetic variants. Furthermore, the fine-mapped genetic variants, confidently located within regulatory regions (comprising 68% of genetic variants), did not significantly co-localize with eQTLs. These findings challenge the hypothesis that most trait-associated variants primarily modulate levels of gene expression. In a separate study, Mostafavi et al. (2023) corroborated the lack of significant colocalization between GWAS hits and eQTLs. Similar to the results of Connally et al. (2022), those of Mostafavi et al. revealed that GWAS hits tend to be situated farther from TSSs than from eQTLs. Furthermore, they conclusively demonstrated that eQTLs and GWAS hits highlight distinct variants and genes. The result of such analysis is that there is no simple link between genetic association and variation in regulatory function (Connally et al. 2022).

## Nonfunctional Binding Sites

Functional binding sites within genomic regions are primarily identified by integrating TF ChIP-seq and RNA-seq [differentially expressed genes (DEGs)] datasets. Target genes are defined as those located near TFBSs detected by chromatin immunoprecipitation, which also exhibit TF-dependent changes in expression. However, the limited proportion of ChIP-seq peaks in the vicinity of DEG datasets suggests that many TFBSs are nonfunctional. In bacteria such as *Escherichia coli*, nonfunctional binding sites (NFBSs) can arise randomly (Mrázek and Karls 2019). In some cases, they were demonstrated to have indirect effects on the effect of the TF they bind to (Gao et al. 2021). Such NFBSs also called latent functional binding sites or cryptic binding sites—(Li et al. 2008, Hajheidari et al. 2019b) are structurally similar to authentic TF motifs and also exhibit a range of binding affinities with TFs, but TF binding at these sites does not elicit transcription. Notably, the abundance of NFBSs greatly surpasses that of functional binding sites. For instance, biophysical investigations focusing on the Egr-1 TF in humans, which targets 9- bp sequences, propose an estimated range of ∼10^6^ to 10^7^ NFBSs within the human genomic DNA, whereas functional binding sites for Egr-1 amount to ∼10^2^ to 10^3^ (Kemme et al. 2016).

NFBSs can be estimated through simple probabilistic calculations in complex organisms such as plants (Kemme et al. 2016). For example, after restricting the analysis to the open chromatin regions of *A. thaliana* [6% of the whole genome (Lu et al. 2019)], the predicted number of NFBSs for the FLC TF is ∼7 × 10^6^. This estimate is based on a recognition site of 21 bp, which includes about 6–12 bp of high-affinity binding sites. In contrast, the count of binding sites for FLC that have an obvious functional effect on downstream genes is markedly lower, amounting to less than ∼10^3^ (Deng et al. 2011, Mateos et al. 2015). The binding of TFs to chromatin is influenced by various physical and biological factors. For instance, nuclear volume significantly affects not only the concentration of TFs but also the concentration of TFBSs and thus the fraction of TFs bound to chromatin. The nuclear diameter in plants ranges from ∼2 µm in meristematic cells (Fal et al. 2021) to 30 µm in epidermal cells (Sakamoto and Takagi 2013). Assuming that nuclei are spherical, the molar concentration of NFBSs of the FLC TF in accessible chromatin regions is ∼10 000 times lower in a nucleus with a diameter of 30 µm (∼2.7 × 10^–7^) than in a nucleus with a diameter of 2 µm (∼2.7 × 10^–3^). This difference results in a higher molar concentration of NFBSs in smaller nuclei, potentially exceeding typical dissociation constants (∼10^−7^ to 10^−12^) for specific or quasi-specific DNA complexes of TFs, suggesting a surplus of NFBSs available for binding compared to functional binding sites (Fujimoto et al. 2000, Kemme et al. 2016, Jin et al. 2021). Such surplus may sequester TFs and function as natural decoys that decrease TF activity.

CCAAT/enhancer-binding protein β (C/EBPα) serve as a prominent example of a TF that can be sequestered away from functional TFBSs by its NFBSs. In mammals, tandem repeats of 171-bp α-satellite DNA located in centromere regions work as NFBSs for C/EBPα. The effective sequestration of C/EBPα molecules during adipocyte differentiation diminishes transcriptional capability and facilitates cell cycle progression. When a mutation alters the binding specificity of C/EBPα, it decreases its affinity for α-satellite DNA while preserving its ability to bind to functional target sites. This results in an increase in C/EBPα binding to promoters, leading to enhanced transcriptional output and ultimately cell cycle exit (Liu et al. 2007). This observation underscores the indirect but nevertheless powerful regulatory role of NFBSs.

**Figure 3.**
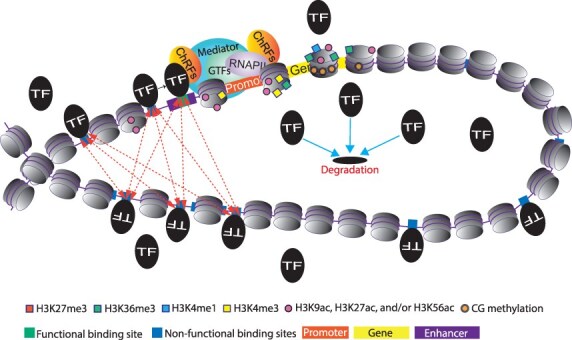
NFBSs and their role in TF regulation. NFBSs potentially influence TF activity through various mechanisms. They may reduce TF availability for their genuine functional binding sites by acting as natural decoys. However, NFBSs may also expedite TFs’ search for a target when they are spatially close to genuine binding sites. Additionally, since DNA-bound TFs are generally more stable than free TFs, natural decoy sites may enhance the protein stability of TFs. Highlighted by dotted arrows are potential paths that NFBSs, particularly those in proximity to TF-specific binding sites, offer for TFs to more efficiently locate their specific functional binding sites. CG methylation depicts DNA methylation in the context of CG dinucleotide. H3K9ac, histone H3 acetylation at lysine 9; H3K27ac, histone H3 acetylation at lysine 27; H3K56ac, histone H3 acetylation at lysine 56; H3K36me3, histone H3 trimethylation at lysine 36; H3K4me1, histone H3 monomethylation at lysine 4; H3K4me3, histone H3 trimethylation at lysine 4; and H3K27me3, histone H3 trimethylation at lysine 27. Histone methylation and acetylation marks are represented by squares and circles, respectively.

Clearly, the potential function of NFBSs can be influenced by the same factors that modulate functional TFBSs. Cell-type-dependent chromatin accessibility, the complex and hierarchical organization of structural components within the cell nucleus, the spatial distribution of target genes for TFs, and the physicochemical properties of TFs that affect both global and local concentrations of TFs and the diversity of their target search patterns all play a role. The function of NFBSs may also change if they occur in close physical proximity with functional TFBSs, because they may ease the search process of TFs for target sites (Fig. 3). Conversely, distant NFBSs are thought to slow down the target search process (Vonhippel and Berg 1989, Tafvizi et al. 2011, Kemme et al. 2016). NFBSs have also been proposed to contribute to TF protein stability (Fig. 3), to reduce the noise around gene expression, or to regulate target genes in a switch-like pattern (Dey and Singh 2021). Furthermore, at least some NFBSs might be genetically redundant with functional binding sites (Spivakov 2014). These sites may help fine tune the expression pattern of genes and developmental robustness (Hajheidari et al. 2019b) and also may become important under special conditions, such as environmental stresses or genetic perturbations (Spivakov 2014). Finally, NFBSs buried in compact chromatin can emerge when environmental changes or genetic perturbations remodel chromatin organization. The widespread presence of NFBSs throughout the genome therefore provides a fertile landscape for the evolution of new transcriptional regulatory circuits, often through a relatively small number of evolutionary events (MacQuarrie et al. 2011). It is tempting to speculate that NFBSs may explain some of the missing link between gene expression and complex trait QTLs and thereby contribute indirectly to the polygenic basis of gene expression variation.

## Are Complex Traits Really Underpinned by Simple Regulatory Variants?

Gene expression variation is believed to be a primary endophenotype explaining variation in higher-level phenotypes (Cookson et al. 2009). Yet, in light of the complexity of gene expression regulation described in this review, one may question whether the genetic basis of gene expression variation is simple. Indeed, the number of positions in the genome that, when altered, can have an effect on gene expression is not reduced to a few *trans*-acting factors and their cognate binding sites. For example, any variant affecting genes that control both global and local DNA dynamics, such as histones, methylases, or demethylases, could alter gene expression via *trans*-acting mechanisms. The specific set of enhancers and silencers that shape the expression of each gene or the stretches of insulating DNA that are needed for the correct positioning of the PIC add to the potential sites in the genome that can contribute to variation in gene expression. Furthermore, the process by which TFs locate their binding sites is inherently probabilistic. Along their search, TFs form weak, transient interactions with numerous DNA regions beyond their specific target sites. These interactions along stretches of open chromatin, play a crucial role in guiding TFs toward their binding sites. As detailed earlier, NFBSs are also likely to further modulate TF activity. The number of positions that, when altered, change a trait is called the mutational target size: the larger the mutational target size, the more variable the trait is expected to be in a population (Landry et al. 2007). If we take into consideration that ultimately transcription is initiated by large protein complexes, which rely on duplicated gene copies, then it becomes clear that, in the genome, the mutational target size of gene expression is large and gene expression could be considered as a polygenic trait as well. If only for the process of TFBS search, gene expression might even form a typical example of a truly omnigenic process (Boyle et al. 2017, Liu et al. 2019, Sinnott-Armstrong et al. 2021, Zhang et al. 2021).

Studies in single-cell organisms like yeast have shown that genetic variation in gene expression arises fast and the regulation of some genes has larger mutational target size than others (Landry et al. 2007). In a more complex multicellular organism like maize, the analysis of allele-specific binding variation of a brassinosteroid-responsive TF in a hybrid (B73xMo17) revealed that as many as 18% of the TF’s target genes display a differently binding *cis*-regulatory element (Hartwig et al. 2023). However, only a quarter of these changes in binding was due to a change in the sequence of a TFBS; most were due to differences in chromatin states (Hartwig et al. 2023). In addition, levels of nonadditive genetic inheritance are substantial for gene expression (Leder et al. 2015, Takou et al. 2022). As a consequence, assuming that the contribution of gene expression variation to complex traits is under the control of a small set of *cis* or *trans* variants might well be inaccurate.

## Conclusion

Major advances in our understanding of the mechanisms controlling gene expression have identified many molecular components that likely interact to determine whether chromatin is open for expression, elucidate how TFs reach their target regulatory regions, and describe how the machinery of transcription is initiated. The complexity we describe reveals that beyond specific TFs and their cognate binding site, the nucleus resembles a complex ecosystem, in which many components interact both directly and indirectly with chromatin and its regulators. Taken together, the molecular work we reviewed indicate that the mutational target size of gene expression is larger than that inferred from the genetics of *cis* and *trans* interaction and the likelihood that variants at these positions interact directly and indirectly is equally large. It is therefore possible that gene expression is also a complex trait, possibly as complex as the higher-level traits they underpin. While this hypothesis remains to be confirmed, we anticipate that considering the polygenic dimension of variation in future studies and models of gene expression will advance our understanding of both complex traits and their potential for adaptation.

## Data Availability

No new datasets were generated in this study.
